# The Women Making Their Mark in Modern Scottish Medical History

**DOI:** 10.3389/jaws.2023.11227

**Published:** 2023-07-03

**Authors:** Stephanie Au, Andrew de Beaux

**Affiliations:** ^1^ Department of General Surgery, Royal Infirmary of Edinburgh, Edinburgh, United Kingdom; ^2^ Edinburgh School of Surgery, University of Edinburgh, Edinburgh, United Kingdom

**Keywords:** gender disparity, diversity, women in surgery, women in medicine, medical history

## Abstract

**Introduction:** Women in medicine and surgery are a recent phenomenon. The aim of this study was to review the modern history of pioneering women in medicine and surgery in Scotland.

**Methods:** A variety of sources were searched including Google, PubMed, and the Royal College of Surgeons of Edinburgh publications to source the material for this paper.

**Results:** Despite over five centuries of Scottish universities offering medical degrees, women have only had the right to study medicine for 150 years. However, the lives of women pioneers who either circumnavigated or surmounted this inequality, namely, “James Barry” and Sophia Jex-Blake, are briefly told.

**Conclusion:** Doctors today owe a debt to those who pushed the boundaries, challenged the unfair rules and tackled institutional gender inequality in medicine. Reading about their lives and work is uplifting.

## Introduction

Scotland is a country that at times led the way in medical education. The King’s College of Aberdeen, now known as the University of Aberdeen, established the first chair of medicine in the English-speaking world in 1497. And in 1505, a group of barber surgeons and other authorities created the organisation now known as the Royal College of Surgeons of Edinburgh.

Back in the 16th century, Henry VIII declared that “No carpenter, smith, weaver or women shall practice surgery.” He forbade women from entering the Company of Barber Surgeons [1]. Indeed, similar edicts were in force across Europe at that time, forbidding women from the study or practice of medicine. Gender disparity has improved remarkably in the past century or so, and there are now many female doctors including surgeons. Yet there is still room for improvement when it comes to diversity in medicine [2]. So how did women first find their way into the field of surgery and medicine in Scotland in modern history?

This paper recounts some of the key women who prevailed against the odds in the practice of medicine and surgery. Space is too limited to tell everyone’s story. Many have lived, worked and influenced the situation in Scotland, with little recognition. But a few names stand out whose remarkable stories ought to be told.

## Methods

Knowledge is a journey, and working in Scotland, some of what we present in this paper we already knew. However, Google, PubMed, and the publications of the Royal College of Surgeons of Edinburgh were searched where necessary to check the accuracy of what is told in this paper, about some remarkable women in recent Scottish History. The search terms were “James Barry AND surgeon,” “The Edinburgh Seven,” and “RCSEd AND women surgeons.” Scientific publications, newspaper and magazine articles were read to add the details and confirm the facts stated in the current paper. The inclusion criteria related to the topic of the current paper, and articles were excluded if they did not add to, or repeated the facts identified. The searches were undertaken during January 2023, by both authors.

## Results and Discussion

### Margaret Bulkley (James Barry)

**FIGURE 1:**
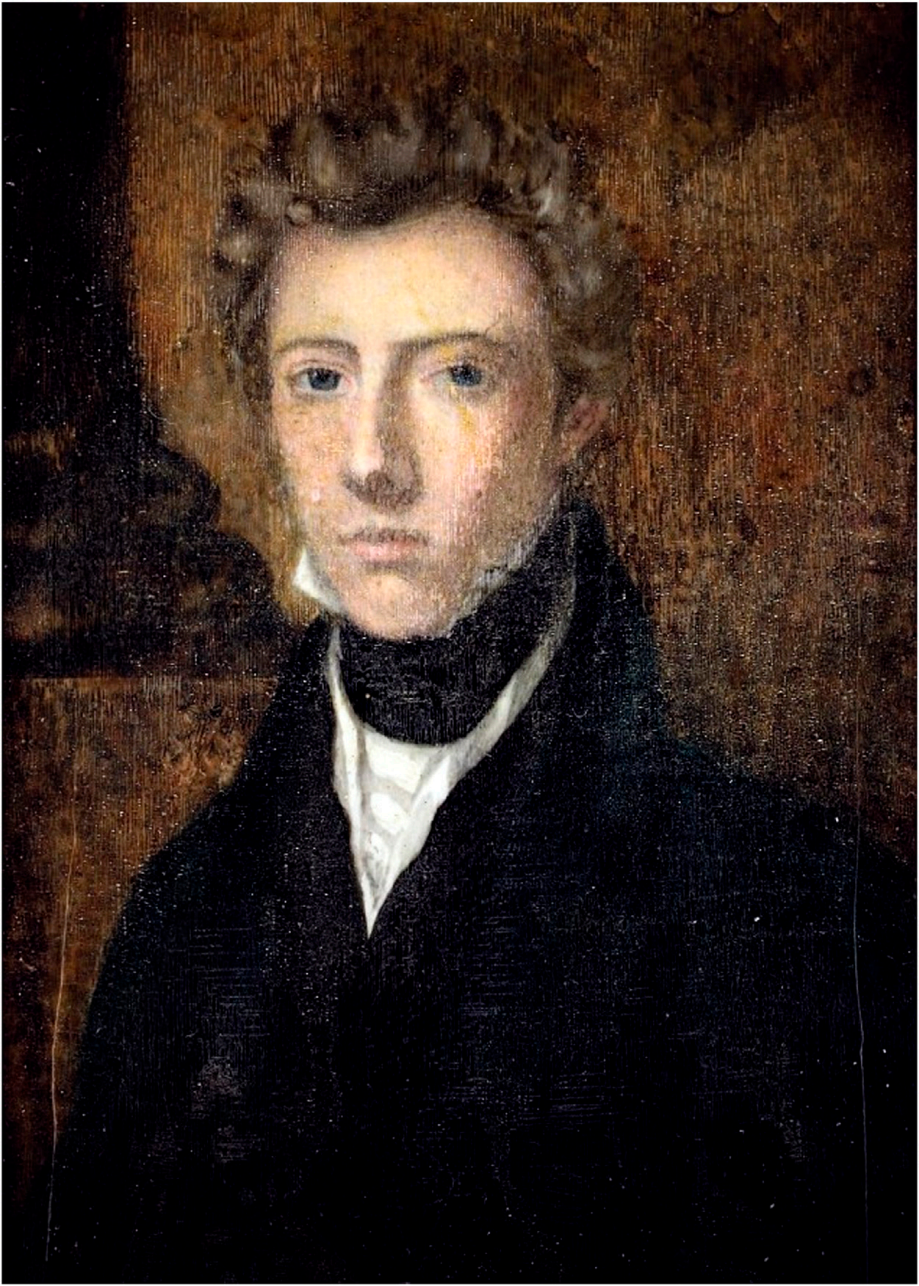
Dr. James Barry, c. 1820s. Photo credit: Oneworld Publications.

The story began in Edinburgh. In the early 19th century, an Irish girl in her late teens named Margaret Bulkley, after failing to obtain a position as a teacher in London, set out to explore a career in medicine. Under the encouragement and help from influential friends of her late uncle, James Barry, an artist and Royal Academician, she received 2 years of further education to prepare herself. However, only men were allowed to enter formal medical education back then. And thus, in November 1809, she disguised herself as a man and travelled by sea to Leith, a port to the north of Edinburgh. She took on the name James Barry, after her late uncle, and enrolled at the University of Edinburgh as a medical student. Despite the startling fact that at that time, only 20% of medical students would graduate from Edinburgh University, she graduated with an M.D. in 1812 [3] (Figure 1).

At that time in the United Kingdom, most surgeons did not go to medical school, but their teaching was an apprentice system straight out of school with older surgeons in their established practices in both charitable public hospitals and private practice. Physicians were educated first in medical school at a university—it was the latter who were called doctors in the United Kingdom, whilst surgeons were called “Mister.” While mister is now a generic title for an adult male, at the time it was originally used by surgeons, it was a title in itself. Skilled craftsmen would be called “Master.” And those that achieved even higher skills in their craft would become “Mister.” The anomaly persists to this day, with male surgeons still referred to as Mr., and female surgeons, irrespective of their marital status as Miss.

That being the case, after her graduation, James Barry travelled to London and became a pupil at the United Hospital of Guy’s and St. Thomas’, where she attended ward teaching and observed surgical procedures. She was examined at the Royal College of Surgeons of England the year after and qualified as a Regimental Assistant.

She continued her disguise and was recruited into the British Army, being posted across the globe from South Africa to Mauritius, Jamaica, Saint Helena, West Indies, Malta, Corfu, and Canada. She was famous for her surgical prowess and was one of the first people who performed Caesarean-sections in which both mother and child survived. During her various posts, she also brought about significant changes to the local population, particularly to the underprivileged. She improved the sanitation and water systems. She improved the conditions and medical care of enslaved people, prisoners and the mentally ill, and established sanctuaries for people suffering from leprosy. During her four decades of military service, she rose through the ranks from Assistant Surgeon to the Forces to ultimately, Inspector General of Hospitals [4].

James Barry eventually retired from the army on 19th July 1859 and died from dysentery on 25th July 1865. It was only upon her death that “James Berry” was discovered to be biologically female by the women who laid out her body [4].

### The Edinburgh Seven

**FIGURE 2:**
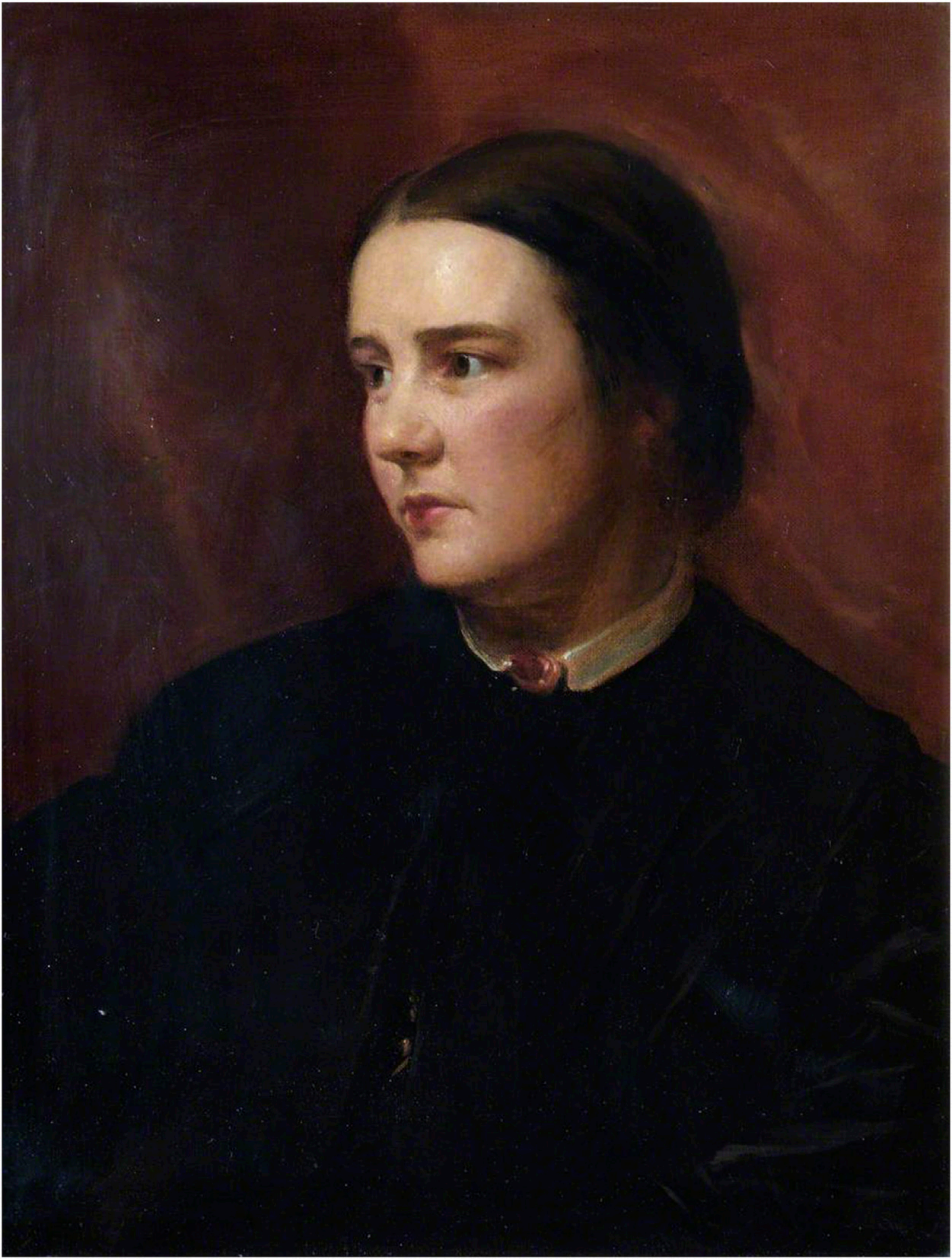
Sophie Jex-Blake. Photo credit: Royal Free Hospital.

The story then takes us back to Edinburgh. Four years after the death of James Barry, Sophia Jex-Blake (Figure 2) applied to study medicine in the University of Edinburgh in March 1869. Her application was rejected by the University Court as the university could not make the necessary arrangements “in the interest of one lady” [5]. More women joined in and the group grew. They were known as the Edinburgh Seven, comprising of Sophia Jex-Blake, Isabel Thorne, Edith Pechey, Matilda Chaplin, Helen Evans, Mary Anderson and Emily Bovell [6]. Their application for matriculation was finally approved by the University Court in the summer of 1869, granting them the right to attend all the classes and examinations required for a degree in medicine, provided that the classes were separate and confined entirely to women [5].

They began preparing for their preliminary examination. Only five of the 152 examination candidates in October 1869 were women but four of the women came in the top seven places. The women signed the matriculation roll in November 1869 [5] and so University of Edinburgh became the first British University to accept women.

However, these women were not being treated as equal to their male counterparts. Edith Pechey ranked first in the Chemistry examination among the students who sat the examination for the first time and therefore had first claim to the Hope Scholarship. However, the scholarship was awarded to male students who achieved lower grades than Pechey as “women are not part of the University class, because they are separately taught” [7].

A debate was held in April 1870 by the University Court to decide whether the women should be allowed in mixed classes, meaning that they would be fully equal to the male students. This would reduce their significantly higher school fees and render them eligible to scholarships and prizes. Some of the prominent professors were against mixed classes and this created a toxic environment that discouraged other staff from teaching them. An increasing number of male students also began to display offensive and often childish behaviours to make the women uncomfortable, including shutting doors in their faces, crowding into seats that they usually occupied and bursting into “horse laughs and howls” whenever the women approached [8].

As antagonism to the Edinburgh Seven grew, this culminated in the Surgeons’ Hall Riot on 18th November 1870. The women were due to sit an Anatomy examination. The street to the examination hall was blocked by a dense mob. As the women battled their way through the crowd, the crowd threw rubbish and mud at them and shouted abuse and insults. When they finally reached the entrance of Surgeons’ Hall, the gates were slammed in their faces. A sympathetic male student eventually came to help and opened the gates for them. This incident however won the women much support from their fellow male students, as they were appalled by the way that the Edinburgh Seven were treated that day [8, 9].

For 2 years, the women diligently studied for the required classes of Chemistry, Practical Chemistry, Institutes of Medicine, Botany, Natural History, Anatomy, Practical Anatomy and Surgery, and passed all the examinations. However, several professors, whose classes the women were required to attend next, refused to teach them as the University regulations permitted but did not expressly require the professors to conduct classes for the women. When they applied to sit for their first professional examination in October 1871, the Medical Faculty rejected their application. On 11th November 1871, the Senatus, with a majority of 1, recommended the University Court to rescind the existing regulations that allowed women to be taught in the University. This decision was supported by the Court of Session in 1873 which ruled that women should not have been admitted in the first place [5].

However, this did not stop the Edinburgh Seven from pursuing their dream of studying medicine. Sophia Jex-Blake moved to London and facilitated the establishment of the London School of Medicine for Women in 1874 [9]. Six of the original seven attended this School. This was also the School that the first English female surgeon, Dame Louisa Aldrich-Blake, graduated from in 1892, earning a gold medal for surgery in the process [10]. She also became the first English women to obtain the degree of Master of Surgery [11].

### Female Pioneers at the Royal College of Surgeons of Edinburgh

The role of women in the Royal College of Surgeons of Edinburgh has a much shorter history. The first women to obtain the FRCSEd diploma was Alice Mabel Headwards-Hunter in 1920. This came shortly after the Sex Disqualification (Removal) Act of 1919 made it illegal in the UK to exclude any woman from employment because of her sex. However, much of Miss Headwards-Hunter’s clinical practice was in India, and her story too is fascinating [12]. The first woman to be elected to the Council of the RCSEd was Miss Caroline May Doig. Her election in 1984, came some 64 years after Miss Headwards-Hunter’s successful diploma by examination. At the start of 2023, four of the 17 Council members are female.

Gone are the times that women needed to disguise themselves as men to enter the field of medicine and surgery. As we celebrated the 150th anniversary of the matriculation of the Edinburgh Seven and awarded them a posthumous MBChB, gender disparity has improved across the field of medicine. Over the recent decade, the number of female doctors has risen by 27% in the United Kingdom, making up just under 48% of all licensed doctors in 2020 [13].

However, general surgery remains a male-dominant specialty with 40% of registrars and just 17% of the consultants being female [14]. It was estimated that general surgery may achieve gender parity at specialty registrars level in the UK by 2028 [14]. Signs of gender bias persist in the surgical field. For example, only 2 of 24 Presidents and 18.1% of the executive committees of surgical societies are women in the United Kingdom [15]. Surgery has a gender pay gap of 21.7% and there are culture barriers to women entering the specialty and perceptions that they would have to adapt their behaviour or expect a less supportive environment [16]. Moreover, it is reported that sexual abuse and harassment are not uncommon in the surgical workplace [17].

## Conclusion

This paper is no more than a whistle-stop tour of the history of women in medicine and surgery. It is hoped that readers are encouraged to research any minorities who shaped medicine and surgery in their own countries. More work needs to be done to understand the reason behind gender disparity and devise policies to attract, recruit and retain women in surgery.
